# Subgroup Behaviors and Factors Influencing Compliance With COVID-19 Preventive Measures Among Undergraduate Students in Southern Thailand

**DOI:** 10.3389/ijph.2024.1606788

**Published:** 2024-09-06

**Authors:** Nonlapan Anujan, Supakorn Sripaew, Pitchayanont Ngamchaliew

**Affiliations:** Department of Family and Preventive Medicine, Faculty of Medicine, Prince of Songkla University, Songkhla, Thailand

**Keywords:** college health, COVID-19, preventive behaviors, latent class analysis, Thailand

## Abstract

**Objective:**

To investigate clusters of students’ COVID-19 preventive behaviors and their associated factors.

**Methods:**

We surveyed undergraduate students using an online questionnaire at a regional university in southern Thailand, between April and June 2022. Statistical analyses included latent class analysis and multinomial regression analysis.

**Results:**

Three latent classes were identified: moderately consistent practitioner (7.5%), high compliance overall (48.9%), and good compliance with routine safeguards (43.6%). Females tended to have high compliance overall (RRR 2.46 95% CI 1.23–4.94), and higher academic performance was associated with high compliance overall and good routine safeguards. Perceived threats from COVID-19 were associated with good compliance with routine safeguards (RRR 4.21 95% CI 1.70–10.45). Benefits of actions and clear cues to action were associated with high overall compliance (RRR 5.24 95% CI 2.13–12.90). Students who perceived feasibility were more likely to be moderately consistent practitioners.

**Conclusion:**

The common clusters of the students’ preventive behaviors were high compliance overall and good compliance with routine preventions. Female, academic performance, perceived threats, and perceived benefits and cues to action were associated with compliance.

## Introduction

Between the recognition of COVID-19 in March 2020 and October 2021, the Coronavirus Disease of 2019 (COVID-19) pandemic had more than two hundred million cases and was attributed to more than four million deaths worldwide [1]. The pandemic had caused significant public health and economic impacts in many countries [2]. COVID-19 is caused by the SARS-CoV-2 virus which can be transmitted from human to human via droplets and aerosols. Transmission is possible despite a person being asymptomatic [3]. After COVID-19 was declared a pandemic, significant efforts were put into dealing with disruptions in health systems and other industries [4]. Global public health sections tackled the spread of COVID-19 by lifting countries’ capacities to screen, treat, and prevent new cases by manipulating population behavior through gradual encouragements and enforcing some new regulations, including wearing face mask in public places [5, 6], things for which it was believed that enforcement alone might not yield effective or sustainable compliance with COVID-19 preventive measures [7].

Since 2020, closures of schools and colleges have been reported worldwide due to pandemic [8]. Reopening universities during the COVID-19 epidemic was a significant challenge in many countries around the world. Israel’s and Korea’s experiences in reopening their universities showed that inadequate implementation of preventive measures could lead to peaks of new cases and reclosures [9, 10]. In Taiwan, there were seven confirmed cases reported from six colleges, of which one university experienced a reclosure 4 months after the pandemic was declared despite having had guidelines for disease surveillance, self-quarantine protocols, a guideline for hygiene practices, ventilation and sanitization control measures, regulations on school gatherings, policies for temporary school closures, and adaptive classes [11].

University students are mostly adolescents [12]. They pay more attention to rewards than to drawbacks and often overlook long-term consequences [13]. In this age group, students prioritize peer effects and behave similarly within their social groups, thus several behaviors can also be clustered [14]. These psychosocial features complicate campaigns which aim to encourage and manipulate the students’ compliance with preventive measures [15]. During COVID-19 pandemic, one study found that these young people were unlikely to report social distancing, washing hands, or avoiding touching faces (38%, 30%, and 30%, respectively) [16]. A Vietnamese study reported that only half (47.4%) had regular hand hygiene practice despite a prevalent favorable attitude to recommended hand hygiene practice (97.9%) [17]. Moreover, the students reported 2.3, 4.0, and 3.7 times lower concerns towards risk of infection, hospitalization, and death from COVID-19, respectively [16].

In late 2021, university teaching in Thailand had to be adapted to control the spread of COVID-19 and was modified to hybrid teaching where in-classroom and video teaching were scheduled alternately. The classes for the social sciences and liberal arts disciplines were held online while the universities tried to keep up with essential laboratory teaching in health science divisions. However, these urgent adaptations might not have sufficed to ensure safe university environments in Thailand during the pandemic. According to Thailand statistics [18], this age group was responsible for 20% of new cases although they had shown favorable attitudes and good compliance with preventive measures [19]. Thus, the Thailand health authorities put a lot of effort into ensuring high vaccine coverage for students before establishing on-site classes, enforcement of strict physical distancing measures, new classroom standards, high compliance with wards hygiene recommendations, and good self-monitoring methods [5, 20].

In the south of Thailand, teaching activities ceased due to the rise in COVID-19 cases in February 2022. Most of the universities decided to reopen their campuses in late June 2022 after the current study was conducted [21]. To control COVID-19 in the university campuses, health authorities demanded good understanding about patterns of behavior that made the students vulnerable to COVID-19. Earlier studies only described the university students’ compliance with individual behaviors and identified certain potential behavior modifiers. However, such simple descriptions might not be sufficient when the students’ preventive behaviors were potentially clustered and influenced by their close ones, including their friends and family members. Therefore, the current study aimed to investigate the underlying clusters of the students’ preventive behaviors in one university, and further identify potential behavioral modifiers of the underlying behavior clusters.

## Methods

### Study Design and Setting

The cross-sectional study was conducted at Prince of Songkla University (PSU), Hat Yai campus between 28 April and 15 June 2022. The data were collected via an online self-reported questionnaire which had been distributed via social media platforms (i.e., the university student affairs page and group emails) and in the form of posters for local advertising. The study did not provide any monetary or other compensation to the participants.

### Study Population and Sample Size Calculation

The participants in our study were undergraduate students of PSU, Hat Yai campus, who could access the internet and understand the Thai language. To estimate required sample size, we calculated the proportions of COVID-19 prevention behaviors in undergraduate students by estimating a finite population proportion [22]. We assumed the compliance with preventive behaviors was 47% and determined the acceptable error at 4.7% [17], which we then adjusted with a design effect of 1.5. Finally, the minimum required sample size was 633 participants.

### Measurements

#### Independent Variables

##### Demographic Characteristics

The study demographic data consisted of age, sex, religion, residence, number of co-residents, sufficiency of monthly budget, faculty group, academic year, academic performance (GPA), mode of study, medical conditions, source of COVID-19 information, source of COVID-19 recommendations, vaccination status, history of COVID-19 infection, and history COVID-19 infections in friends and family. Age was classified into two groups: 18–21 years, and over 21 years. Residence was defined as the students’ current place of living, categorized into two groups: dormitory, and home. The sufficiency of the monthly budget as perceived by the respondent was divided into three groups: insufficient, sufficient, and having savings. The faculty was divided into three groups based on their learning activities during the pandemic: health sciences (hospital-based), sciences and technology (laboratory-based), and social sciences and others (online-based). Academic performance was measured by grade point average (GPA) of the previous year, which was later categorized into three groups: high, moderate, and low.

##### Perceptions Related With COVID-19 and Preventive Measures

We developed the study questionnaires incorporating factors influencing youth behaviors from previous studies [14, 15]. Perceptions are important internal mechanisms that can influence one’s compliance with health recommendation [23], thus we hypothesized the perceptions were the main exposure in the study. The students’ perceptions were measured by a 4-point Likert scale questionnaire with responses ranging from strongly disagree to strongly agree. An item-objective congruency index (IOC) was used to evaluate the questions in the questionnaire by three experts. The IOC was 0.96–1.00 for each item, and 0.91 overall. We conducted exploratory factor analysis (EFA) to assess the latent constructs of the students’ perceptions. Principal axis factoring with varimax rotation was used, and we found three variables: perceptions towards threats from COVID-19 (including one’s own perceived susceptibility to develop COVID-19 and perceived severity of COVID-19), perceived benefits of following recommended measures and cues to the actions, and the feasibility of complying with the recommended actions (defined as the degree of confidence individuals felt in their ability to adopt and maintain the recommended preventive measures) [24]. The questionnaire was internally consistent (alpha = 0.61 to 0.89 for each domain, and 0.77 overall). The total scores in each perception domain were later categorized into low and high levels by the group median (25, 14, and 27 for perceived threats from COVID-19, perceived feasibility of compliance with recommended measures, and perceived benefits of following recommended measures and cues to the actions, respectively).

#### Dependent Variables – COVID-19 Preventive Behaviors

We formulated ten behavior measurement questions based on local guidelines and CDC recommendations [5]. The questions asked participants to rate their frequency of seven preventive measures: appropriate hand hygiene practices, cough etiquette, mask-wearing, avoiding crowds, social distancing, cleaning contact surfaces in daily life, and self-health monitoring. Responses were based on a 4-level frequency scale ranging from 1 to 4 (1 = never, 2 = rarely, 3 = sometimes, and 4 = often). Compliance with COVID-19 preventive behaviors was represented by the proportion of participants in each frequency category. The IOCs were acceptable (ranging from 0.67 to 1 for each item and 0.97 overall), and the items were internally consistent (alpha = 0.79).

### Statistical Analysis

The compliance with each preventive behavior was described in frequency and percentage. We evaluated possible participant-centered clusters of behavior from the ten preventive behaviors by latent class analysis (LCA) following standard recommendations [25]. Our analysis evaluated one to six-classes model and incorporated social factor covariates: gender, sufficiency of monthly budget, year of study, and place of residence. Participants with incomplete covariate data were removed from the LCA. The Chi-square test was used to examine differences in the distributions of each factor among the classes. Subsequently, we chose variables with *p* < 0.05 to be factors for adjusting effect estimates in the regression analysis. Relative Risk Ratios (RRR) with 95% confidence intervals (95% CI) were acquired from multivariable multinomial regression. The Akaike information criterion (AIC), Bayesian information criterion (BIC), maximum log-likelihood, and entropy were used as diagnostic parameters for choosing a proper model in terms of the number of classes, together with its interpretability. R software version 4.2.0 with ggplot2, latticeExtra, poLCA, epicalc, and nnet were used in the analysis.

## Results

### Participant Characteristics

Of the 687 participants who had complete data (97.6% of the total sample), more than half were female (58.8%), with most aged between 18 and 21 years old (89.5%), and Buddhist (79.8%). The majority of the participants stayed at home (73.5%), the highest number had 4-6 members in the same residence (48.5%), and had sufficient monthly budget and had savings (42.6%). The majority of the participants were studying in the science and technology group (60.8%), almost half were studying in the 2nd academic year (49.3%), and had moderate grades (46.6%). Nearly ninety percent of the did all their academic work online. Only one-fifth of the participants had medical condition (23.3%), of which the most common was allergic rhinitis. Around half of the participants were feasible students, while less than half had high perceived threats from COVID-19 and had high perceptions of the benefits of following the recommended behaviors and cues to action. The baseline characteristics of the participants are reported in Table 1.

**TABLE 1:** The three behavioral classes with associated variables (participants who had complete data = 687) (Subgroup Behaviors and Factors Influencing Compliance With COVID-19 Preventive Measures Among Undergraduate Students in Southern Thailand, Thailand, 2022).

Variable	Behavioral patterns of participants			Total participants (N, %)	*P*-value*
	High compliance overall (N, %)	Moderately consistent practitioner (N, %)	Good compliance with routine safeguards (N, %)		
Age group					0.411
18–21 years	308 (90.6)	48 (92.3)	259 (87.8)	615 (89.5)	
More than 21 years	32 (9.4)	4 (7.7)	36 (12.2)	72 (10.5)	
Sex					<0.001
Female	211 (62.1)	16 (30.8)	177 (60)	404 (58.8)	
Male	129 (37.9)	36 (69.2)	118 (40)	283 (41.2)	
Religion					0.01
Buddhist	287 (84.4)	38 (73.1)	223 (75.6)	548 (79.8)	
Islam	53 (15.6)	14 (26.9)	72 (24.4)	139 (20.2)	
Residence					0.561
Dormitory	86 (25.3)	12 (23.1)	84 (28.5)	182 (26.5)	
Home	254 (74.7)	40 (76.9)	211 (71.5)	505 (73.5)	
Number of co-residents					0.333
1–3 people	154 (45.3)	21 (40.4)	140 (47.5)	315 (45.8)	
4–6 people	170 (50)	25 (48.1)	138 (46.8)	333 (48.5)	
More than 6 people	16 (4.7)	6 (11.5)	17 (5.8)	39 (5.7)	
Perceived monthly budget sufficiency					0.001
Insufficient	43 (12.6)	11 (21.2)	76 (25.8)	130 (19.0)	
Sufficient	142 (41.8)	18 (34.6)	104 (35.3)	264 (38.4)	
Having savings	155 (45.6)	23 (44.2)	115 (39)	293 (42.6)	
Faculty group					<0.001
Health sciences	93 (27.4)	2 (3.8)	68 (23.1)	163 (23.7)	
Science and technology	211 (62.1)	45 (86.5)	162 (54.9)	418 (60.8)	
Social sciences and other	36 (10.6)	5 (9.6)	65 (22)	106 (15.5)	
Academic year					0.384
1	35 (10.3)	6 (11.5)	30 (10.2)	71 (10.3)	
2	173 (50.9)	19 (36.5)	146 (49.5)	338 (49.3)	
3	98 (28.8)	20 (38.5)	78 (26.4)	196 (28.5)	
More than 3	34 (10)	7 (13.5)	41 (13.9)	82 (11.9)	
Academic performance					<0.001
Low (GPA <3)	87 (25.6)	28 (53.8)	65 (22)	180 (26.2)	
Moderate (GPA 3–3.5)	147 (43.2)	18 (34.6)	155 (52.5)	320 (46.6)	
High (GPA >3.5)	106 (31.2)	6 (11.5)	75 (25.4)	187 (27.2)	
Mode of study					0.184
Online	304 (89.4)	48 (92.3)	252 (85.4)	604 (87.9)	
Onsite	36 (10.6)	4 (7.7)	43 (14.6)	83 (12.1)	
Had any medical condition					0.146
Yes	89 (26.2)	13 (25.0)	58 (19.7)	160 (23.3)	
No	251 (73.8)	39 (75.0)	237 (80.3)	527 (76.7)	
Source of COVID-19 information					0.758
1–3 media	196 (57.6)	31 (59.6)	163 (55.3)	390 (56.8)	
More than 3 media	144 (42.4)	21 (40.4)	132 (44.7)	297 (43.2)	
Source of COVID-19 recommendations					0.05
Family	72 (21.2)	16 (30.8)	59 (20)	147 (21.4)	
Government	85 (25)	11 (21.2)	77 (26.1)	173 (25.2)	
Healthcare provider	166 (48.8)	19 (36.5)	127 (43.1)	312 (45.4)	
Friends and other	17 (5)	6 (11.5)	32 (10.8)	55 (8.0)	
Complete vaccination^a^					0.479
Yes	286 (84.1)	45 (86.5)	258 (87.5)	589 (85.7)	
No	54 (15.9)	7 (13.5)	37 (12.5)	98 (14.3)	
History of self-COVID-19 infection					0.149
Yes	74 (21.8)	13 (25.0)	84 (28.5)	171 (24.9)	
No	246 (78.2)	39 (75.0)	211 (71.5)	516 (75.1)	
History of friend COVID-19 infection					0.887
Yes	257 (75.6)	39 (75.0)	218 (73.9)	514 (74.8)	
No	63 (24.4)	13 (25.0)	77 (26.1)	173 (25.2)	
History of family COVID-19 infection					0.305
Yes	150 (44.1)	27 (51.9)	146 (49.5)	323 (47.0)	
No	170 (55.9)	25 (48.1)	149 (50.5)	364 (53.0)	
Perceived threats from COVID-19					<0.001
Yes	143 (42.1)	7 (13.5)	144 (48.8)	294 (42.8)	
No	177 (57.9)	45 (86.5)	151 (51.2)	394 (57.2)	
Perceived feasibility of compliance with recommended measures					<0.001
High	173 (50.9)	43 (82.7)	164 (55.6)	380 (55.3)	
Low	147 (49.1)	9 (17.3)	131 (44.4)	307 (44.7)	
Perceived benefit of following recommended measures and cues to the actions					<0.001
High	199 (58.5)	7 (13.5)	105 (35.6)	311 (45.3)	
Low	121 (41.5)	45 (86.5)	190 (64.4)	376 (54.7)	

* Chi-square test.

^a^ Received at least two consecutive doses of the same vaccine.

Most participants reported high compliance with wearing a mask in public settings or crowds (89.5%) and monitoring their health daily (72.3%). Over half of the participants often washed their hands during daily activities (59.1%), used public transportation only as needed (58.7%), covered their mouth when coughing or sneezing (54%), and avoided touching the front of their mask (52.8%). One-third of the participants often avoided crowds and poorly ventilated spaces (40.5%), washed their hand after coughing or sneezing (36.4%), cleaned high-touch surfaces before using public areas (34.2%), and maintained a 1–2 m distance from others (34.1%). The variations of the participants’ COVID-19 preventive behaviors are shown in Table 2.

**TABLE 2:** Compliance with recommended COVID-19 preventive behaviours (Total participants = 704) (Subgroup Behaviors and Factors Influencing Compliance With COVID-19 Preventive Measures Among Undergraduate Students in Southern Thailand, Thailand, 2022).

COVID-19 preventive behaviour	Frequency (N, %)			
	Often	Sometimes	Rarely	Never
Performing hand washing during daily activities	416 (59.1)	242 (34.4)	43 (6.1)	3 (0.4)
Performing hand washing after coughing	256 (36.4)	319 (45.3)	112 (15.9)	17 (2.4)
Cough etiquette	380 (54.0)	218 (31.0)	77 (10.9)	29 (4.1)
Wearing mask in public places	630 (89.5)	62 (8.8)	11 (1.6)	1 (0.1)
Avoiding touching outside surface of the mask	372 (52.8)	258 (36.6)	60 (8.5)	14 (2.0)
Avoiding crowded places	285 (40.5)	311 (44.2)	91 (12.9)	17 (2.4)
Limit public transport usage	413 (58.7)	183 (26.0)	66 (9.4)	42 (6.0)
Maintaining 1–2 m distance from others	240 (34.1)	338 (48.0)	120 (17.0)	6 (0.9)
Cleaning contact surfaces	241 (34.2)	310 (44.0)	130 (18.5)	23 (3.3)
Self-health monitoring	509 (72.3)	156 (22.2)	34 (4.8)	5 (0.7)

**FIGURE 1:**
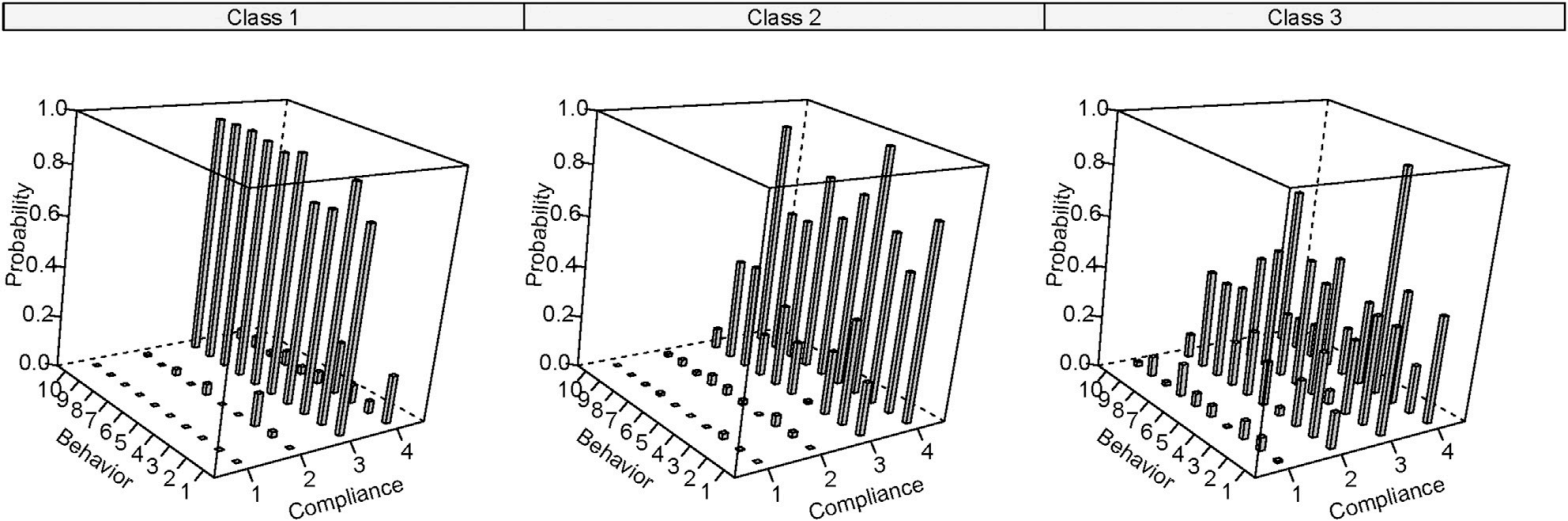
Latent classes of recommended coronavirus disease 2019 preventive measures in the participants (Subgroup Behaviors and Factors Influencing Compliance With COVID-19 Preventive Measures Among Undergraduate Students in Southern Thailand, Thailand, 2022). Remarks: class1 = moderately consistent practitioner, class 2 = high compliance overall, class 3 = good compliance with routine safeguards, compliance score 1 = rarely practiced, 2 = sometimes practiced, 3 = often practiced, 4 = regularly practiced, and probability represents conditional probability of levels of compliance based on the latent class, behavior numbers from one to ten represent performing hand washing in daily life (1), performing hand washing after coughing (2), cough etiquette (3), wearing mask in public places (4), avoiding touching outside surface of the mask (5), avoiding crowded places (6), limit public transport usage (7), maintaining 1–2 meter distance from others (8), cleaning contact surfaces (9), and self-health monitoring (10), respectively.

**FIGURE 2:**
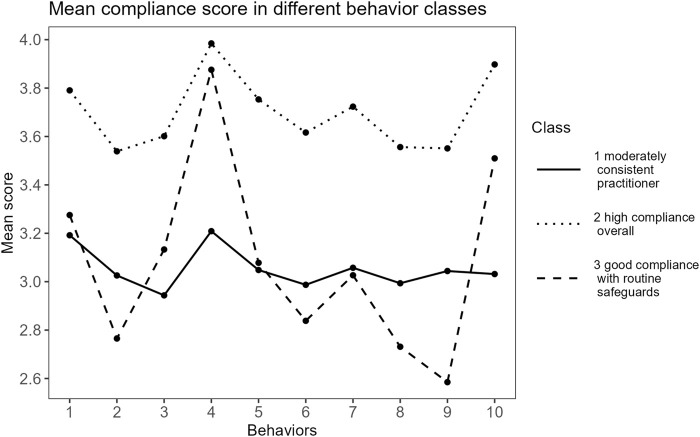
Mean compliance scores in different behavior classes (Subgroup Behaviors and Factors Influencing Compliance With COVID-19 Preventive Measures Among Undergraduate Students in Southern Thailand, Thailand, 2022). Remarks: *y*-axis is partly collapsed to illustrate variations of the score, behavior numbers from one to ten represent performing hand washing in daily life (1), performing hand washing after coughing (2), cough etiquette (3), wearing mask in public places (4), avoiding touching outside surface of the mask (5), avoiding crowded places (6), limit public transport usage (7), maintaining 1–2 m from others (8), cleaning contact surface (9), and self-health monitoring (10), respectively.

We constructed a three-class model which was statistically and conceptually suitable to capture latent subpopulations (Figure 1). The details of the diagnostic statistics used in all the models and their values are provided in Supplementary Table S1. Figure 1 shows the conditional probability for each response (compliance score in each behavior) in each latent class. The model predicted that classes one, two and three would share 7.5%, 48.9%, and 43.6% probability of the study sample. The mean scores of each behavior in each latent class were also used to describe the features of the clusters (Figure 2). Moderately consistent practitioner (class 1) and high compliance overall (class 2) represented clusters of individuals who had moderately high and high mean scores across the ten behaviors, respectively, and good compliance with routine safeguards (class 3) referred to the cluster of people who had high compliance in only certain routine measures (often the measures applied in public places) while their compliances with some actions were relatively low.

Table 1 illustrates the overall characteristics of the participants and across the latent classes. We found that sex, religion, sufficiency of monthly budget, faculty group, academic performance (GPA), high perceived threats from COVID-19, high perceived feasibility of compliance with recommended measures, and high perceived benefits of following recommended measures and cues to the actions significantly differed among the three classes (*p* < 0.05).

We used multinomial regression analysis to identify factors associated with COVID-19 preventive behaviors (Table 3). Our results showed that females were more likely to have high compliance overall compared with males (RRR 2.46, 95% CI 1.23–4.94). Participants with a moderate GPA were more likely to be categorized as good compliance with routine safeguards and high compliance overall (RRR 3.22, 95% CI 1.59–6.54 and RRR 2.05, 95% CI 1.01–4.16, respectively) while a high GPA was more likely to indicate a participant with high compliance overall (RRR 3.15, 95% CI 1.14–8.74). Participants with high perceived threats from COVID-19 were more likely to have good compliance with routine safeguards (RRR 4.21, 95% CI 1.70–10.45). Additionally, participants with a high perceived feasibility of compliance with recommended measures were less likely to have good compliance with routine safeguards and high compliance overall (RRR 0.28, 95% CI 0.12–0.62 and RRR 0.30, 95% CI 0.14–0.68, respectively). Individuals who highly perceived benefits of following recommended measures and cues to the actions were somewhat likely to have high compliance overall (RRR 5.24, 95% CI 2.13–12.90).

**TABLE 3:** Factors associated with COVID-19 preventive behaviors (reference = moderately consistent practitioner, N = 687) (Subgroup Behaviors and Factors Influencing Compliance With COVID-19 Preventive Measures Among Undergraduate Students in Southern Thailand, Thailand, 2022).

Variable	RRR (95% CI)	
	Good compliance with routine safeguards	High compliance overall
Sex (ref. = Male)		
Female	1.85 (0.92–3.72)	2.46 (1.23–4.94)*
Religion (ref. = Buddhist)		
Islam	1.01 (0.48–2.14)	0.70 (0.33–1.51)
Monthly budget sufficiency (ref. = insufficient)		
Sufficient	0.85 (0.35–2.05)	2.28 (0.92–5.65)
Having savings	0.56 (0.23–1.33)	1.53 (0.62–3.77)
Faculty group (ref. = health sciences)		
Science and technology	0.25 (0.05–1.12)	0.29 (0.06–1.35)
Social sciences and other	0.48 (0.08–2.79)	0.21 (0.04–1.23)
Academic performance [ref. = low (GPA <3)]		
Moderate (GPA 3–3.5)	3.22 (1.59–6.54)*	2.05 (1.01–4.16)*
High (GPA >3.5)	2.69 (0.97–7.50)	3.15 (1.14–8.74)*
Perceived threats from COVID-19 (ref. = low)		
High	4.21 (1.70–10.45)*	2.10 (0.84–5.24)
Perceived feasibility of compliance with recommended measures (ref. = low)		
High	0.28 (0.12–0.62)*	0.30 (0.14–0.68)*
Perceived benefit of following recommended measures and cues to the actions		
High	1.49 (0.60–3.70)	5.24 (2.13–12.90)*

RRR, relative risk ratio; CI, confidence interval, * = *p* < 0.05, AIC =1,118.26.

## Discussion

Our study was exploratory research aimed at examining the prevalence of compliance with preventive measures and the factors associated with COVID-19 preventive behaviors among undergraduate students at a large university in Southern Thailand. At the time of the study, the university was emphasizing various COVID-19 preventive policies, including campaigning for students to adhere to several preventive measures, implementing electronic temperature checkpoints in most public areas, and conducting online surveillance of COVID-19 through daily self-reported symptoms [20]. Most of the study participants reported high compliance with wearing masks in public places and self-health monitoring. The high compliance with mask-wearing in our study was similar to findings from studies in Vietnam and Malaysia [26]. However, care is required to ensure good mask-wearing compliance during outdoor public activities. Previous studies of undergraduate students in the US showed that mask-wearing in outdoor spaces was relatively low compared with indoors [27, 28]. We also found that only one-third of the participants maintained good hand hygiene, regularly cleaned contact surfaces, and practiced proper physical distancing. These low compliance rates, however, were not uncommon. Similar findings have been reported at other universities in Asia and the United States [17, 29–31]. Therefore, university authorities should consider these commonly low compliance rates as areas for improvement when preparing for similar outbreaks in the future. Nevertheless, extra care should be taken to balance physical distancing with opportunities for healthy social interactions, as excessive distancing could negatively impact students’ mental health and increase the risk of depression [32].

For health behaviors, investigating multiple separate behaviors and trying to modify these behaviors separately might not be an efficient method for a university as the high risk students often present with a cluster of health-risk behaviors [14]. Latent class analysis is an approach to identify unmanifested groups of behaviors could provide insights into behavior modifications for an outbreak control for COVID-19 or other respiratory viral infections. We classified the COVID-19 preventive behaviors into three classes: moderately consistent practitioners (class 1), high overall compliance (class 2), and good compliance with routine safeguards (class 3). Classes 2 and 3 made up approximately 93 percent of the study sample. The behavior patterns in these two classes were similar in terms of having high mask wearing and self-monitoring compliances, which could further imply that a majority of the participants had relatively equivalent awareness towards these two behaviors compared to the other eight practices. However, the lower compliances in class 2 could reflect the needs for additional health promotion campaigns.

We identified several factors associated with good compliance, including female gender, a high GPA, a high perceived threat from COVID-19, a high perceived feasibility of compliance with preventive measures, and a high perceived benefit of following recommended measures and cues to action. Females were more likely to have high overall compliance. Several previous studies have shown that females exhibited higher adherence to COVID-19 preventive measures [33–35]. Studies explained that females had higher conscientiousness and agreeableness [36], and also had higher interests in health information, thus they were more compliant to the COVID-10 prevention [37]. Apart from gender, lower student GPAs were associated with an increased risk of low prevention compliances, similar to its association with other public health recommendations [38]. In addition, the extent to which students perceived threats was particularly associated with compliance with routine safeguards. Leveraging awareness of threats from COVID-19 could be an effective strategy to promote compliance with mask-wearing and self-monitoring. Furthermore, the perceived benefits of following recommended measures and cues to action were significant motivators for high overall compliance. Several studies have demonstrated a strong positive relationship between perceived benefits, cues to action, and the adoption of COVID-19 preventive behaviors among undergraduates in Thailand and many other countries [39–43]. Therefore, policy should aim at promoting awareness of the benefits of COVID-19 preventive measures and providing resources as cues to action, with a particular focus on male students and those with a low GPA.

Undergraduate students are mostly adolescents and study under structured coursework [44]. There are interventions to promote the student behaviors through persuasive techniques and social engineering [45]. The persuasive approach [46] utilizes simple, credible, relevant, and emotional messages connected to the actual pandemic situation and distributes these messages through student online social networks or coursework panels. Social engineering involves various socio-environmental modifications, such as rule enforcement or enhancing accessibility to face masks and hand sanitizers throughout the university [47]. Nevertheless, we identified perceived feasibility as a risk factor for poor routine (class 2) and overall (class 3) hygiene practices. This phenomenon could be due to a paradoxical effect: while perceiving abundant feasibility of carrying out preventive behaviors initially increases motivation to adhere to the measures, excessive resources can reverse this effect [48]. Thus, monitoring students’ behaviors should be conducted in parallel with these promotions.

### Strengths

Our study had several strengths. We applied local COVID-19 prevention recommendations with world standards in our instrument’s design to enhance the study’s internal validity. The internet-based survey also helped us to achieve a good response rate during the pandemic time and reduce biased responses from students who were concerned about whether their behaviors were socially acceptable. In addition, we applied latent class analysis to further identify potential clusters of students’ preventive behaviors to illustrate the existence of clusters where students had similar behaviors rather than using simple descriptive statistics. This empirical evidence could help policymakers identify potential groups of students and prioritize actions to tackle certain behavioral risk factor issues.

### Limitations and Further Study Suggestions

There were certain limitations in our study. First, we conducted the study during a period of stable pandemic situation and a small re-surge of COVID-19 case numbers. The dynamics of COVID-19 might have influenced the students’ awareness through surging pandemic information and a wide range of regulations at the participants’ residences. Thus, caveats should be considered that the presented associations might have been confounded by the intensity of the outbreak. Second, our latent class analysis was based on the behaviors of the study population. This could limit the generalizability of the classes and their associated factors to the other populations. Third, our data were based on a undergraduate students who had the capacity to access to the internet. Caution is advised when using the behavioral patterns for non-undergraduates and those who cannot access online resources, as perceptions could be altered significantly by the receipt of information showing individual’s susceptibility to the active infection.

### Implications

During a respiratory virus outbreak, university authorities should consider campaigns to promote hand hygiene, cough etiquette, environment cleaning, and balanced physical distancing for the university students while routine safeguards are regulated. In addition, policy or campaign designs should pay special attention to groups less likely to follow the suggestions of authorities, including low GPA and male students. They should also increase awareness of the incoming threats, highlight the benefits of preventive actions, and exhibit clear cues to the suggested actions, to promote high compliance with preventive measures in their students.

### Conclusion

The study highlighted a relatively low compliance with physical distancing among undergraduate university students. The most common clusters of the undergraduate students’ preventive behaviors were high compliance overall, and good compliance only to some routine preventions. Female students and participants who had better academic performance tended to have high compliance for prevention recommendations in general. The perceptions towards threats from COVID-19, and perceived benefits and cues to the preventive actions, were potential behavioral modifiers which require attention from the university.
