# Supplementary material for: Subgroup Behaviors and Factors Influencing Compliance With COVID-19 Preventive Measures Among Undergraduate Students in Southern Thailand
Source: Int J Public Health. 2024 Sep 6;69:1606788. doi: 10.3389/ijph.2024.1606788 (PMC11412799; doi:10.3389/ijph.2024.1606788)
Supplement: Supplementary file 1 [file DataSheet1.docx]

**Supplementary data 1. The questionnaires**

Perceived threats questionnaire

1. You have some chance of getting COVID-19 infection.
2. You are more likely to get a COVID-19 infection than people in middle age.
3. Due to the COVID-19 situation, you need to adapt your lifestyle, such as increasing your mask-wearing and washing your hands more often.
4. Even after being vaccinated, it is possible to get COVID.
5. COVID-19 can spread through droplets when a patient is coughing, sneezing, or touching.
6. Most COVID-19 patients are asymptomatic or have mild symptoms which could be self-limiting without the need of antiretroviral medications.
7. There could be a case in which you bring COVID-19 to ones close to you, including friends and family members.
8. If you get COVID-19, it is more likely that it can be treated at home or in a community quarantine center, rather than requiring hospitalization.

Perceived feasibility questionnaire

1. Wearing a face mask makes your friends see you as a person who is panicking about a COVID-19 infection.
2. You do not need to wear a mask in open public spaces because the ventilation is good enough.
3. Frequent hand washing interferes with your daily routines or work activities.
4. Hand sanitizers and alcohol-based hand sanitizers are expensive and hard to find.
5. Cleaning surfaces of common equipment in public places after every single usage is troublesome.

Perceived benefits and cues to action questionnaire

1. Physical distancing reduces the spread of infection to your family and friends.
2. Hand washing is an important activity you should always do before touching your own face.
3. Cleaning high-touch areas such as doorknobs, tables, and counters with detergent or soap and water on a regular basis can help reduce the spread of COVID-19.
4. Daily health monitoring allows you to preliminarily screen if you have ax COVID-19 infection.
5. Recommendations from healthcare providers enable you to follow COVID-19 prevention measures more effectively.
6. Your family members have advised you to always perform COVID-19 precautions.
7. Your friend (s) has always exhibited regular COVID-19 prevention behaviors thus they are a good model for you.
8. You would be more cautious when someone close to you is diagnosed with COVID-19.

COVID-19 preventive behaviors questionnaire

1. Hand washing by soap or alcohol gel during daily activities
2. Hand washing by soap or alcohol gel after coughing or sneezing
3. Covering your mouth with a tissue or the inside of your elbow when coughing or sneezing
4. Wearing a mask in public settings or crowds
5. Avoiding touching the outside of the mask
6. Avoiding crowded and poorly ventilated spaces
7. Using public transportation only if needed
8. Maintaining 1-2 meter distance from others
9. Cleaning high-touch surfaces before using certain equipment in public areas
10. Monitoring your health daily

**Supplementary table 1. Latent class analysis for classifying COVID-19 preventive behavior patterns**

| Model | Maximum LL | AIC | BIC | Entropy |
| --- | --- | --- | --- | --- |
| 1 class | -6520.869 | 13101.74 | 13237.71 | 9.491804 |
| 2 class | -6065.145 | 12268.29 | 12581.02 | 8.884503 |
| 3 class | -5856.649 | 11929.3 | 12418.79 | 8.599983 |
| 4 class | -5715.781 | 11725.56 | 12391.82 | 8.367119 |
| 5 class | -5667.152 | 11706.3 | 12549.32 | 8.3024 |
| 6 class | -6446.51 | 13343.02 | 14362.79 | 9.486442 |

Maximum entropy = 13.86294
